# A Novel Lipoate-Protein Ligase, Mhp-LplJ, Is Required for Lipoic Acid Metabolism in *Mycoplasma hyopneumoniae*

**DOI:** 10.3389/fmicb.2020.631433

**Published:** 2021-01-18

**Authors:** Jin Jin, Huan Chen, Ning Wang, Kemeng Zhu, Huanhuan Liu, Dongfang Shi, Jiuqing Xin, Henggui Liu

**Affiliations:** ^1^State Key Laboratory of Veterinary Biotechnology, Harbin Veterinary Research Institute, Chinese Academy of Agricultural Sciences, Harbin, China; ^2^Department of Preventive Veterinary Medicine, College of Veterinary Medicine, Northeast Agricultural University, Harbin, China; ^3^Department of Biology, Guangdong Provincial Key Laboratory of Cell Microenvironment and Disease Research, Shenzhen Key Laboratory of Cell Microenvironment and SUSTech-HKU Joint Laboratories for Matrix Biology and Diseases, Southern University of Science and Technology, Shenzhen, China; ^4^College of Life Science, Yangtze University, Kingchow, China

**Keywords:** lipoate-protein ligase, lipoic acid, *Mycoplasma hyopneumoniae*, dihydrolipoamide dehydrogenase, lipoic acid analogs

## Abstract

Lipoic acid is a conserved cofactor necessary for the activation of several critical enzyme complexes in the aerobic metabolism of 2-oxoacids and one-carbon metabolism. Lipoate metabolism enzymes are key for lipoic acid biosynthesis and salvage. In this study, we found that *Mycoplasma hyopneumoniae* (*M. hyopneumoniae*) Mhp-Lpl, which had been previously shown to have lipoate-protein ligase activity against glycine cleavage system H protein (GcvH) *in vitro*, did not lipoylate the lipoate-dependent subunit of dihydrolipoamide dehydrogenase (PdhD). Further studies indicated that a new putative lipoate-protein ligase in *M. hyopneumoniae*, MHP_RS00640 (Mhp-LplJ), catalyzes free lipoic acid attachment to PdhD *in vitro*. In a model organism, Mhp-LplJ exhibited lipoate and octanoate ligase activities against PdhD. When the enzyme activity of Mhp-LplJ was disrupted by lipoic acid analogs, 8-bromooctanoic acid (8-BrO) and 6,8-dichlorooctanoate (6,8-diClO), *M. hyopneumoniae* growth was arrested *in vitro*. Taken together, these results indicate that Mhp-LplJ plays a vital role in lipoic acid metabolism of *M. hyopneumoniae*, which is of great significance to further understand the metabolism of *M. hyopneumoniae* and develop new antimicrobials against it.

## Introduction

*Mycoplasma hyopneumoniae* (*M. hyopneumoniae*) is the causative agent of enzootic pneumonia (EP) and is widespread in pig herds (Mulcock, 1965; Maes et al., 1996). Despite the low direct mortality, immunosuppression caused by *M. hyopneumoniae* and secondary infections by other pathogens greatly increase morbidity and mortality (Yu et al., 2018). Increasing evidence indicates that *M. hyopneumoniae* infection is associated with porcine respiratory disease complex (PRDC), although the mechanisms of PRDC caused by *M. hyopneumoniae* are barely understood (Maes et al., 2008; Jeffrey et al., 2012). In addition, EP increases production costs due to vaccination and medication, but the effects of prevention and treatment of this disease are barely satisfactory (Takeuti et al., 2017). Some studies have shown that current commercial vaccines only slow down *M. hyopneumoniae* transmission and are unable to prevent colonization of *M. hyopneumoniae* and the development of lung lesions (Matthijs et al., 2019). In terms of drug therapies, the persistence of *M. hyopneumoniae* is observed after antibiotic treatments, although the pathogen is sensitive to these antibiotics *in vitro* (Carrou et al., 2006). Therefore, it is imperative to develop new vaccines and medicines.

Lipoic acid [(R)-5-(1,2-dithiolane-3-yl) pentanoic acid, 6,8-dithiooctanoic acid, thioctic acid] is an organosulfur cofactor that is required for the function of several key enzymatic complexes in central metabolism (Cao et al., 2018b; Lavatelli et al., 2020). The lipoate-dependent enzymatic complexes include α-ketoacid dehydrogenase complexes [pyruvate dehydrogenase (PDH), 2-oxoglutarate dehydrogenase (OGDH), branched-chain 2-oxoacid dehydrogenase (BCOADH)], acetoin dehydrogenase (AoDH) and the glycine cleavage system (Gcs) (Spalding and Prigge, 2010; Cronan, 2016). In contrast to most cofactors, lipoic acid is functional only when covalently bound to lipoyl domains (LDs) via an amide linkage. The lipoyl-lysine arm formed between the lipoic acid carboxyl group and the conserved lysine ε-amino group of LDs is required to shuttle intermediates between the active sites of the multienzymatic complexes (Perham, 2000).

**FIGURE 1:**
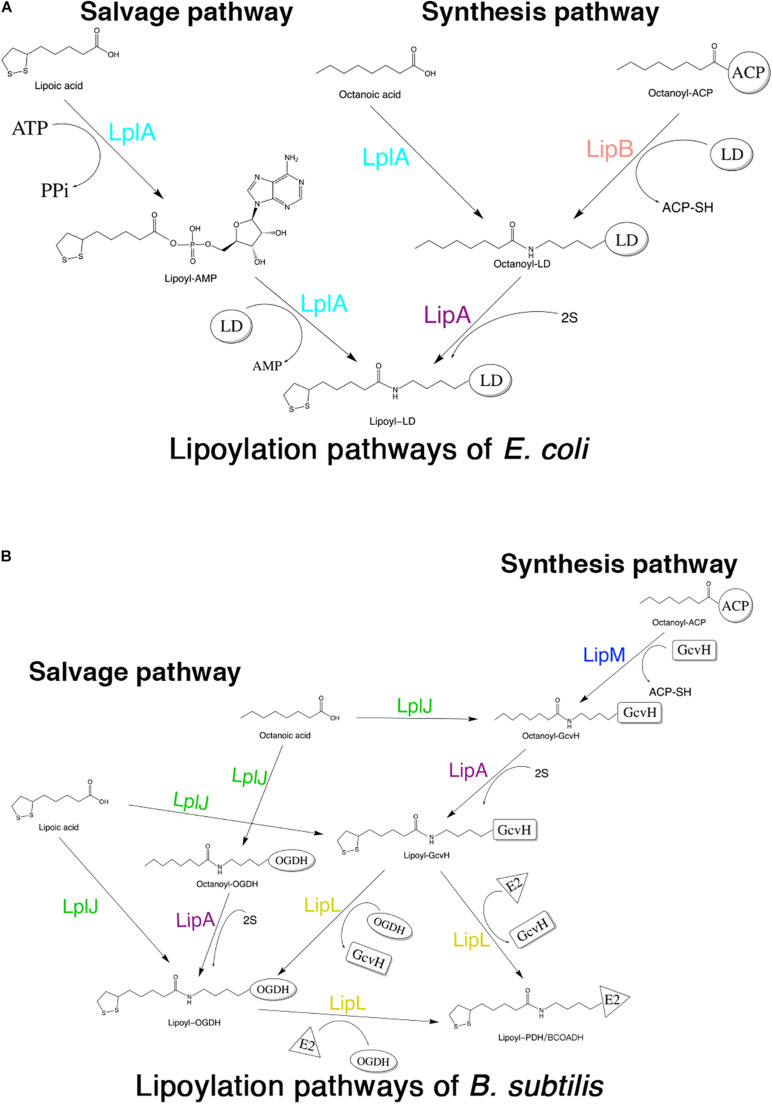
Schematic diagram of lipoic acid metabolism pathways in *E. coli* and *B. subtilis*. Both *E. coli* and *B. subtili*s have two independent pathways for lipoic acid metabolism. **(A)** Lipoylation pathways of *E. coli*. LplA is able to transfer both free lipoic acid as well as free octanoic acid to the apo-proteins in the salvage pathway. LipB and LipA are involved in the lipoic acid synthesis pathway of *E. coli*. LD: lipoly domain of lipoate-dependent protein. **(B)** Lipoylation pathways of *B. subtili*s. In the salvage pathway, LplJ can only modify GcvH and OGDH, and that LipL is necessary to lipoylate the E2 subunits of PDH and BCOADH (represented by E2). In the synthesis pathway, first, LipM specifically transfers octanoate from ACP to GcvH. Then, LipA catalyzes sulfur insertion to form lipoyl-GcvH and LipL transfers the lipoyl group from GcvH to other lipoate-requiring subunits. The lipoyl-OGDH could also serve as a lipoate donor for the lipoylation of PDH and BCOADH.

Although lipoic acid as a cofactor has been known in bacterial metabolism for more than 50 years, the pathways of its biosynthesis in different microorganisms have not been elucidated until recent years (Cao et al., 2018c). The enzymes involved in lipoic acid metabolism are highly conserved in different organisms; however, the mechanisms by which these enzymes function are different among organisms. The lipoate metabolic pathways of *Escherichia coli* (*E. coli*) and *Bacillus subtilis* (*B. subtilis*) are the best understood (Cao et al., 2018a). Both of these organisms have two pathways for lipoic acid metabolism, the salvage pathway and the synthesis (or *de novo*) pathway (Morris et al., 1995; Christensen et al., 2011b). In the salvage pathway, lipoate-protein ligases [lipoate-protein ligase A (LplA) in *E. coli* (*E. coli* LplA) and lipoate-protein ligase J (LplJ) in *B. subtilis* (*B. subtilis* LplJ)] use exogenous lipoic acid in a two-step reaction (Cronan et al., 2005; Martin et al., 2011) (Figures 1A,B). *E. coli* LplA has lipoate and octanoate ligase activities for all lipoate-requiring subunits. While *B. subtilis* LplJ has a tight substrate specificity as it is only capable of transferring free lipoic acid and octanoic acid to GcvH and E2 subunit of OGDH (Rasetto et al., 2019). In the synthesis pathway of *E. coli*, octanoyltransferase (LipB) transfers the octanoyl moiety from octanoyl-ACP (acyl carrier protein), and then lipoyl synthase (LipA) catalyzes sulfur insertion to form a lipoyl moiety (Miller et al., 2000; Jordan and Cronan, 2003) (Figure 1A). However, in the lipoic acid synthesis pathway of *B. subtilis*, glycine cleavage system H protein (GcvH) is used as an intermediate carrier. Lipoyl-[GcvH]:protein *N*-lipoyltransferase (LipL) catalyzes the transamidation of the lipoyl moiety from lipoyl-GcvH to other subunits; prior to this reaction, octanoyltransferase (LipM) specifically modifies the octanoyl of GcvH with octanoyl-ACP as the donor (Christensen et al., 2011b). After that, LipA is responsible for the insertion of sulfur atoms, as in *E. coli* (Martin et al., 2009). LipL can catalyze the transfer of lipoyl moiety of lipoyl-OGDH to E2 subunits of PDH and BCOADH (Figure 1B) (Rasetto et al., 2019).

In mycoplasma species, lipoic acid metabolism is poorly understood. We previously reported a lipoate-protein ligase, Mhp-Lpl, in *M. hyopneumoniae* (Zhu et al., 2020). However, we found Mhp-Lpl is not able to modify dihydrolipoamide dehydrogenase (PdhD), a lipoate-requiring protein of *M. hyopneumoniae*. According to this phenomenon, we identified a new lipoate-protein ligase in *M. hyopneumoniae*. Based on the model organism *E. coli*, this enzyme shows both lipoate and octanoate ligase activities for PdhD. We also further confirmed that the lipoic acid analogs 8-bromooctanoic acid (8-BrO) and 6,8-dichlorooctanoate (6,8-diClO) can inhibit the lipoylation of PdhD catalyzed by Mhp-LplJ and the growth of *M. hyopneumoniae in vitro* to different degrees.

## Materials and Methods

### Materials

All chemicals used in this study were purchased from Sigma-Aldrich unless stated otherwise. Antibodies were obtained from Abcam (rabbit anti-lipoic acid antibody, goat anti-mouse secondary antibody, HRP conjugated) and LI-COR (IRDye 680RD goat anti-mouse secondary antibody, IRDye 800CW goat anti-rabbit secondary antibody). PCR amplification was performed using *Taq* (New England Biolabs) PrimeSTAR HS (Premix) (TaKaRa) polymerase. Restriction enzymes and T4 DNA ligase were supplied by TaKaRa. A ClonExpress II One Step Cloning Kit was purchased from Vazyme. GE Healthcare provided a Ni-NTA agarose column.

### Constructed Plasmids, Mutant Bacterial Strains, and Culture Conditions

The bacterial strains and plasmids used in this study are listed in Table 1. The *E. coli* strains MG1655, DH5α, and BL21(DE3) were used for genome mutation, DNA manipulation and protein expression, respectively. The vectors pET32a and pET22b were used to express recombinant proteins in BL21(DE3), while pET-HITES was gifted by Qiang Li of Tsinghua University and used to express apo lipoate-requiring substrates in the MG1655-derived strain DE0626. The plasmid pBAD322G was gifted by John Cronan of University of Illinois and used in complementation assays. The plasmids pKD3, pKD46, and pCP20 were used for disrupting chromosomal genes in MG1655.

**TABLE 1:** Strains and plasmids used in this work.

Strain/Plasmid	Relevant characteristics	Source or ref.
**Strain**		
DE0626	MG1655 Δ*lplA* Δ*lipB*	Christensen and Cronan, 2009
RE0403	MG1655 Δ*aceF::pdhD* Δ*lplA* Δ*lipB*	This study
RE1009	MG1655 Δ*aceF::pdhD* Δ*lplA* Δ*lipB* Δ*gcvH*	This study
RE1011	MG1655 Δ*aceF::pdhD* Δ*lplA* Δ*lipB* Δ*lipA*	This study
*M. hyopneumoniae* J	Attenuated strain	Lab stock
*M. hyopneumoniae* 168-L	Attenuated strain	Lab stock
**Plasmid**		
pET-HITES	Host-independent T7 expression system, Kn^R^	Liang et al., 2018
pBAD322G	Low copy expression vector, Gm^R^	Cronan, 2006
pKD3	Template plasmid containing FRT-flanked cat, Amp^R^	Datsenko and Wanner, 2000
pKD46	Red recombinase expression plasmid, Amp^R^	Datsenko and Wanner, 2000
pCP20	*FLP*^+^, λ cI857^+^, Rep^ts^, Amp^R^ Cm^R^	Datsenko and Wanner, 2000
pEA001	pET32a encoding *M. hyopneumoniae* PdhD	This study
pX1	pET32a encoding Mhp-Lpl	Zhu et al., 2020
pX3	pET32a encoding *E. coli* LplA	Zhu et al., 2020
pEB001	pET22b encoding Mhp-LplJ	This study
pEB002	pET22b encoding Mhp-LplJ_G77A_	This study
pEB003	pET22b encoding Mhp-LplJ_G77S_	This study
pEB004	pET22b encoding Mhp-LplJ_K131A_	This study
pEB005	pET22b encoding Mhp-LplJ_K131S_	This study
pEH001	pET-HITES encoding apo *M. hyopneumoniae* GcvH	This study
pEH002	pET-HITES encoding apo *M. hyopneumoniae* PdhD	This study
pEH003	pET-HITES encoding apo *E. coli* GcvH	This study
pEH004	pET-HITES encoding apo *E. coli* SucB	This study
pEH005	pET-HITES encoding apo *E. coli* AceF	This study
pBG001	pBAD322G encoding *E. coli* LplA	This study
pBG002	pBAD322G encoding *E. coli* LipB	This study
pBG003	pBAD322G encoding Mhp-LplJ	This study
pBG004	pBAD322G encoding Mhp-Lpl	This study

All plasmids used and constructed in this study are shown in Table 1. The primers used are shown in Table 2. The *M. hyopneumoniae pdhD* gene and *Mhp-lplJ* were synthesized with the TGA stop codons in the open reading frames (ORFs) replaced with TGG. The synthesized *pdhD* was amplified with the primer pair pA1 and inserted into pET32a between the *Nde*I and *Not*I sites to obtain the plasmid pEA001. The synthesized *Mhp-lplJ* was amplified with the primer pair pB1 and inserted into pET22b between the *Sal*I and *Not*I sites to obtain the recombinant plasmid pEB001. The Mhp-LplJ_G__77__A/S_ and Mhp-LplJ_K__131__A/S_ mutations were generated by site-directed mutagenesis using the primer pairs MJ77A, MJ77S, MJ131A, and MJ131S, respectively.

**TABLE 2:** Primers used in this research.

Primer name		Sequence(5′-3′)
pA1	F R	CGCCATATGTATAAATTTAAATTTGCTGA TTGCGGCCGCTTTAATTTTCTTCAATTTTG
pB1	F R	GCGTCGACGAGATAATTATGAAAATTTA TTGCGGCCGCAACTCCAAAAATTGTATCTA
MJ77A	F R	AGGGGGTGCGGCGGTCTATCATGATCTA GAAAGCCGACGGTAGATTTCAATAT
MJ77S	F R	AGGGGGTTCAGCGGTCTATCATGATCTA GAAAGCCGACGGTAGATTTCAATAT
MJ131A	F R	CAGCAATTTCGGGTAATGCCCAGAT CGCCATTGACAATTAAATCATTTCG
MJ131S	F R	CAAGTATTTCGGGTAATGCCCAGAT CGCCATTGACAATTAAATCATTTCG
pH1	F R	gcgaggagctgttcaccgggATGAAGAAGATCGCAAATTTCCTG gactctagcactaagcggccgCTA**GTGGTGGTGGTGGTGGTG**AAAATCTTCCAGCTCATCA
pH2	F R	gcgaggagctgttcaccgggATGTATAAATTTAAATTTGCTGATATCGG gactctagcactaagcggccgCTA**GTGGTGGTGGTGGTGGTG**TTTAATTTTCTTCAATTTTG
pH3	F R	gcgaggagctgttcaccgggATGAGCAACGTACCAGCAGAACT gactctagcactaagcggccgCTA**GTGGTGGTGGTGGTGGTG**CTCGTCTTCTAACAATGCT
pH4	F R	gcgaggagctgttcaccgggATGAGTAGCGTAGATATTCTGGTCCC gactctagcactaagcggccgCTA**GTGGTGGTGGTGGTGGTG**CACGTCCAGCAGCAGACG
pH5	F R	gcgaggagctgttcaccgggATGGCTATCGAAATCAAAGTACCG gactctagcactaagcggccgCTA**GTGGTGGTGGTGGTGGTG**CATCACCAGACGGCGAAT
pG1	F R	ttgggctagcaggaggaattcATGTCCACATTACGCCTGCT tccgccaaaacagccaagcttCTACCTTACAGCCCCCGCCATCCAT
pG2	F R	ttgggctagcaggaggaattcATGTATCAGGATAAAATTCTTGTCCG tccgccaaaacagccaagcttTTAAGCGGTAATATATTCGAAGTCCG
pG3	F R	ttgggctagcaggaggaattcATGGAGATAATTATGAAAAT tccgccaaaacagccaagcttAACTCCAAAAATTGTATCTA
pG4	F R	ttgggctagcaggaggaattcATGTACCTGATTGAACCGAAAC tccgccaaaacagccaagcttTTCAGCAGCAGGTTACAAAT
DE*lplA*	F R	aagagtgacccattactacaagaaaggaaatcgttAGCGATTGTGTAGGCTGGAG caaatcgaagagaaagttgcccgcatgggcgggtaaTTAACGGCTGACATGGGAATTAG
DE*lipB*	F R	tcccccacttttactcattctccacggagatgccgttAGCGATTGTGTAGGCTGGAG tgacccagtgtaaattgggccattgatgtatggaaTTAACGGCTGACATGGGAATTAG
DE*lipA*	F R	ctttccttcgtaattcgcaactggaacacgcacgctAGCGATTGTGTAGGCTGGAG gttttttatcagacagatgtaagtaattattacaggaTTAACGGCTGACATGGGAATTAG
DE*gcvH*	F R	gccgtcgcgtgatttacttttttggagattgattgAGCGATTGTGTAGGCTGGAG accctgatcctctcccgcagaagaggaataaagccgTTAACGGCTGACATGGGAATTAG
RE*aceF*1	F R	agttaacccgcgtctggcgtaagaggtaaaagaataATGTATAAATTTAAATTTGCTGATATCGG ctccagcctacacaatcgctTTATTTAATTTTTTCAATTTTG
RE*aceF*2	F R	AGCGATTGTGTAGGCTGGAGcagaaaaaagccggccgttgggccggctcttttacTTAACGGCTGACATGGGAATTAG

*Lowercase letters indicate homologous arms for recombinant methods. Histidine labels are in bold, and restriction sites are underlined.*

The gene fragments of *M. hyopneumoniae gcvH*, *M. hyopneumoniae pdhD*, *E. coli gcvH*, *E. coli sucB*, and *E. coli aceF* were inserted into pET-HITES between the *Bse*RI and *Eag*I sites after amplification with primer pairs pH1–pH5 from the corresponding genomic DNAs. These plasmids, named pEH001–pEH005, were used to express apo lipoate-requiring substrates. *E. coli lplA*, *E. coli lipB, Mhp-lplJ*, and *Mhp-lpl* were amplified with the primer pairs pG1–pG4 and ligated into pBAD322G between the *Eco*RI and *Hin*dIII sites to obtain plasmids pBG001–pBG004, respectively.

All *E. coli* mutants were derivatives of *E. coli* MG1655 and were constructed by the method reported by Datsenko and Wanner (2000). The *lplA* gene was replaced with a chloramphenicol resistance cassette by transformation of PCR products generated with the primer pairs DE*lplA*. The resistance gene was removed using FLP recombinase encoded by the temperature-sensitive plasmid pCP20. The genes *lipB*, *lipA*, and *gcvH* were deleted by transformation of PCR products generated with the primer pairs DE*lipB*, DE*lipA*, and DE*gcvH*, respectively. To construct a strain in which the *aceF* gene of *E. coli* is replaced with the wild-type *pdhD* gene of *M. hyopneumoniae*, a fragment containing the intact *pdhD* fused with an antibiotic cassette and the homologous region of *aceF* was amplified by overlap PCR with the primer pairs RE*aceF*1 and RE*aceF*2. All the mutant strains are described in Table 1.

*Mycoplasma hyopneumoniae* was cultured in mycoplasma medium (BasalMedia, China) containing 20% (v/v) swine serum for 6–7 days at 37°C. *E. coli* strains were grown in Luria-Bertani (LB) rich or M9 minimal medium (Miller, 1972). Antibiotics were used at the following concentrations: chloramphenicol, 40 μg/mL; ampicillin sodium, 100 μg/mL; kanamycin sulfate, 50 μg/mL; and gentamicin sulfate, 50 μg/mL. L-arabinose was used at a final concentration of 0.2%. Lipoic acid was added at a final concentration of 1 mM.

### Protein Expression and Purification

To express and purify the hexa-histidine-tagged *E. coli* LplA, Mhp-Lpl, Mhp-LplJ, Mhp-LplJ mutants and holo-PdhD, the recombinant plasmids pX3, pX1, pEB001-005, and pEA001 were transformed into *E. coli* BL21 (DE3) cells. The cells were cultured at 37°C in Luria broth. When the OD_600_ reached 0.5, the cells were induced to express the target proteins with 1 mM isopropyl 1-thio-β-D-galactopyranoside (IPTG) at 25°C for 15 h. The cells were collected by centrifugation and resuspended in lysis buffer (50 mM Tris-HCl, 300 mM NaCl, 1 mM dithiothreitol and 20 mM imidazole, pH 8.0). Following sonication, crude lysates were centrifuged at 12,000 *g* for 30 min to remove the cell debris. The supernatants were applied to purify the recombinant proteins with a Ni-NTA-agarose affinity chromatography column according to the instructions. The purified proteins were concentrated by ultrafiltration (3- or 10-kDa cutoff), and concentrations were measured by extinction coefficients and spectrophotometric determination. Protein purity was monitored by SDS-PAGE. To obtain apo lipoate-proteins, the plasmids pEH001–pEH005 were transformed into *E. coli* lipoic acid auxotroph strain DE0626. Protein expression and purification were performed as described above.

### Production of a Monoclonal Antibody in Mice

Specific monoclonal antibodies against *M. hyopneumoniae* PdhD were produced by hybridoma technology. Briefly, 6- to 8-week-old female mice were immunized with purified protein emulsified with complete or incomplete Freund’s adjuvant. After three immunizations with an interval of 2 weeks, the mice were sacrificed to isolate spleen cells. Splenocytes were then fused with mouse SP2/0 myeloma cells using PEG 1450. The resulting hybridoma cells were plated onto ten 96-well plates and selected with hypoxanthine-aminopterin-thymidine (HAT)-conditioned medium for 2 weeks. The antibodies specific for *M. hyopneumoniae* PdhD in the supernatant from each well were detected by ELISA. The positive hybridoma cells were harvested and injected into the abdominal cavities of the mice to produce monoclonal antibodies (mAbs). The animal experiment was approved by the Institutional Animal Care and Use Committee of Harbin Veterinary Research Institute (IACUC#181122-02, approval date: 22 November 2018).

### Structural Modeling and Molecular Docking

Conservation analysis of Mhp-LplJ amino acid residues was based on Clustal Omega (Fábio et al., 2019). Homology modeling predicting the three-dimensional (3D) structure of Mhp-LplJ was conducted using Swiss-Model (Jürgen and Torsten, 2006; Waterhouse et al., 2018). Sequence alignment was performed using ClustalX, and amino acid residues were colored by the ESPript server (Xavier and Patrice, 2014). Molecular docking analyses of Mhp-LplJ were performed using lipoic acid or lipoyl-AMP as ligands to study the mode of interaction within the binding site. The 3D structures of lipoic acid and lipoyl-AMP were obtained from the Protein Data Bank (PDB) database. PyMOL, AutoGrid, and Autodock Tools (version 1.5.6) were used during the molecular docking analysis. LigPlot^+^ was used to generate 2D ligand-protein interaction diagrams.

### Ligation Assays *in vitro*

Lipoate ligation reactions (100 μL) contained 50 mM sodium phosphate (pH 7.0), 5 mM disodium ATP, 5 mM dithiothreitol, 1 mM MgCl_2_, 1 mM lipoic acid, 20 μM apo-substrate, and 4 μM lipoate-protein ligase. After incubation at 37°C for 3 h, the lipoylation of the indicated proteins was analyzed by SDS-PAGE followed by western blot using rabbit anti-lipoic acid antibodies (Anti-LA). To analyze the active sites of Mhp-LplJ, Mhp-LplJ_G__77__A/S_, or Mhp-LplJ_K__131__A/S_ mutants were added to the reaction as the lipoate-protein ligase. The effect of 8-BrO and 6,8-diClO on Mhp-LplJ lipoylation activity was assessed by adding analogs dissolved in dimethylsulfoxide (DMSO) at a final concentration of 1 mM. The reaction was carried out under the conditions described above using apo-PdhD as a substrate. Octanoate ligation assay was performed as lipoate ligation assay except free octanoic acid was added to reactions instead of lipoic acid. The results were analyzed by gel shift assay.

### Gel Shift Assay and LC-MS/MS for PdhD Modification Analysis

The reaction (100 μL) contained 50 mM sodium phosphate (pH 7.0), 5 mM disodium ATP, 1 mM MgCl_2_, 1 mM lipoic acid, 20 μM apo-PdhD and 4 μM Mhp-LplJ. After incubation at 37°C for 3 h, the proteins in the reaction system were loaded on an 8% native polyacrylamide gel and separated by electrophoresis.

Nano-LC-LTQ-Orbitrap XL MS/MS was performed to detect lipoate modification of PdhD. Chymotrypsin and trypsin-digested peptides were separated by a C_18_ reversed-phase column (filled with 3 μm ReproSil-Pur C_18_-AQ from Dr. Maisch GmbH) and loaded by a C_18_ reversed-phase column (filled with 5 μm ReproSil-Pur C_18_-AQ from Dr. Maisch GmbH) onto the nanoLC-LTQ-Orbitrap XL system (Thermo). Data were analyzed by Proteome Discoverer (version 1.4.0.288, Thermo Fischer Scientific). The MS2 spectra were searched in the PdhD sequence plus Contaminants (cRAP) database using the SEQUEST search engine. Lipoylation of lysine and oxidation of methionine were set as variable modifications. The matching of searched peptide and MS spectra was filtered by Percolator calculation.

### Cell-Based Lipoate Ligation Assay

*Mycoplasma hyopneumoniae* genes were inserted into the arabinose-inducible plasmid pBAD322G and transformed into *E. coli* lipoic acid auxotroph strain DE0626 (Christensen and Cronan, 2010). To prevent carryover lipoic acid, all plasmid-carrying strains were grown for 36 h in M9 minimal medium containing 5 mM acetate, 5 mM succinate, 0.4% glycerol and appropriate antibiotics to bypass the lipoic acid-requiring aerobic pathways. Strains were then grown for 2 days on M9 minimal plates with or without supplementation of lipoic acid.

To analyze the lipoate modification of the indicated protein in the cells complementary with different genes, the *E. coli*-derived strain DE0626/RE0403 was transformed with a plasmid expressing a candidate lipoate-protein ligase (pBG001–pBG004). Transformants were grown in 5 mL Luria broth at 37°C for 16 h with or without lipoic acid. Cells were harvested by centrifugation and resuspended in 0.5 mL PBS. The total proteins of the cells were analyzed by western blot as described for the lipoate ligation assay.

### Western Blot Analysis

Western blot assays were carried out using the following procedure. Briefly, the samples were loaded and separated on a 12% SDS-polyacrylamide gel and transferred by electrophoresis to nitrocellulose (NC) membranes (Millipore) for 30 min at 25 V. The membranes were first blocked with TBS buffer (100 mM Tris base and 0.9% NaCl, pH 7.4) containing 0.1% Tween 20 and 5% non-fat milk powder. Then, they were washed three times and probed for 1 h with Anti-LA (1:8000) or mouse anti-PdhD monoclonal antibodies (Anti-PdhD) (1:10000) for 1 h. Next, the membranes were analyzed by using Odyssey CLx Image Studio software after incubation for 1 h with an IRDye 680RD goat anti-mouse secondary antibody or IRDye 800CW goat anti-rabbit secondary antibody (1:10000).

### Surface Plasmon Resonance

The binding kinetics between Mhp-LplJ and lipoic acid or analogs were analyzed in real time by surface plasmon resonance (SPR) on a Biacore 8K machine with CM5 chips (GE Healthcare) at room temperature. Mhp-LplJ was diluted to 50 μg/mL with 10 mM acetate (pH = 5.0) and immobilized through a standard amine-coupling protocol with an amine coupling kit (GE Healthcare). The molecules (lipoic acid, 8-BrO and 6,8-diClO) were serially diluted using PBS-P buffer (GE Healthcare) containing 5% DMSO. The analytes passed through chip surface flow cell 1 (fc1, activate/deactivate during immobilization) and flow cell 2 (fc2, immobilized by Mhp-LplJ) at a rate of 30 μL/min. The response units (RU) were measured in real-time and are shown in the sensorgram. Association phases were monitored for 120 s, dissociation phases were monitored for 300 s, and the experiments were performed in 5% DMSO PBS-P buffer consisting of 20 mM phosphate, 2.7 mM KCl, 137 mM NaCl, 0.005% (*v/v*) P20 and 5% (*v/v*) DMSO. The results were analyzed using Biacore Insight Evaluation Software.

### Assay of *M. hyopneumoniae* Growth Inhibition by Lipoic Acid Analogs

*Mycoplasma hyopneumoniae* strain J was cultured in the medium described above at 37°C and 100 rpm for 6 days. The seed liquid was diluted 100-fold into fresh medium containing an indicated final concentration of analogs dissolved in DMSO or the same volume of DMSO. After incubation at 37°C for 10 days, the copy titers of all *M. hyopneumoniae* samples were determined by a color changing units (CCU) assay (Stemke and Robertson, 1990; Calus et al., 2010).

## Results

### PdhD Is the Lipoylated Protein in *M. hyopneumoniae*

**FIGURE 2:**
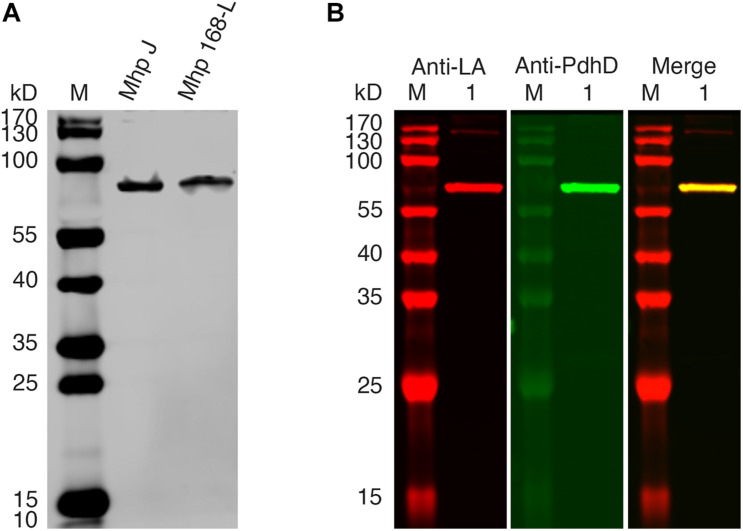
PdhD is the lipoylated subunit in *M. hyopneumoniae*. **(A)** *M. hyopneumoniae* strain J and strain 168-L were analyzed by western blot using Anti-LA. **(B)** PdhD expressed in the *M. hyopneumoniae* J strain was probed with Anti-LA and Anti-PdhD. Lane M is the molecular weight ladder. Lane 1 is the cell lysate of the *M. hyopneumoniae* J strain.

PDH, OGDH, BCOADH, AoDH, and Gcs are the common lipoate-dependent enzymes in bacteria, but we failed to identify the genes encoding these enzymes except PDH and Gcs in the genome sequence of *M. hyopneumoniae*. To determine the lipoylated protein in *M. hyopneumoniae*, the total proteins of the cells from *M. hyopneumoniae* strain J and strain 168-L were analyzed using a western blot assay with Anti-LA. As shown in Figure 2A, a distinct band appeared in the scanned NC membrane. To identify this lipoylated protein in *M. hyopneumoniae*, the band in the gel was excised and analyzed by LC-MS, and the results indicate that the lipoylated protein might be the PdhD encoded by *pdhD* (Supplementary File S1). To further confirm this conclusion, Anti-PdhD was produced, and the PdhD protein expressed in *M. hyopneumoniae* J strain was analyzed using a western blot assay with both Anti-LA and Anti-PdhD simultaneously. As shown in Figure 2B, PdhD was finally verified to be the lipoylated protein in *M. hyopneumoniae*.

### A New Putative Lipoate-Protein Ligase Exists in *M. hyopneumoniae*

**FIGURE 3:**
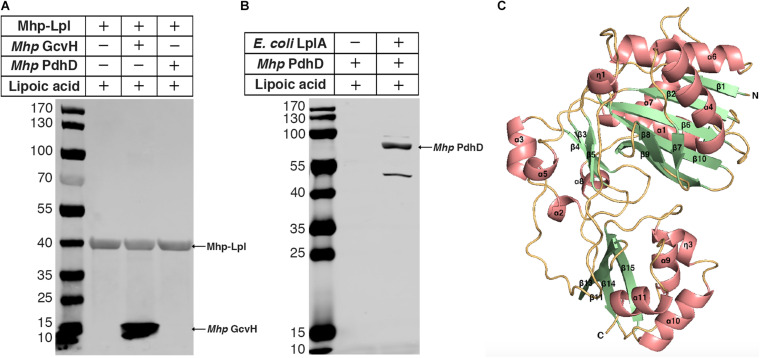
Modification analysis of PdhD by Mhp-Lpl and structural modeling of Mhp-LplJ. **(A)** The lipoate ligase activity of Mhp-Lpl was tested using the lipoate ligation assay with GcvH (*Mhp* GcvH) and PdhD (*Mhp* PdhD) of *M. hyopneumoniae* as substrates. Following the reaction, the lipoate modification of the substrates was probed with Anti-LA using western blot assay. **(B)** The lipoate modification of PdhD was probed with Anti-LA after reaction with *E. coli* LplA using western blot assay. **(C)** The overall structure of apo-Mhp-LplJ. Salmon, α-helix; pale green, β-sheet; light orange, random coil. The 3D structure model of apo-Mhp-LplJ was predicted using the Swiss-Model server with Sp-Lpl (PDB: 1VQZ) as the template.

Previously, we reported that Mhp-Lpl catalyzes the attachment of lipoic acid to the lipoyl domains of GcvH *in vitro* (Zhu et al., 2020). We wondered whether the lipoate modification of PdhD is catalyzed by Mhp-Lpl. However, analysis showed that Mhp-Lpl failed to catalyze the lipoylation of PdhD *in vitro* (Figure 3A). The functions of lipoate-protein ligases among organisms are highly conserved. As *E. coli* LplA could catalyze lipoic acid attachment to the dihydrolipoyllysine-residue acetyltransferase component of PDH (AceF), a similar lipoate-dependent substrate as PdhD in *E. coli*, it is theoretically confirmed for *E. coli* LplA to catalyze the lipoylation of PdhD if purified PdhD retains its functional structure. Therefore, we used *E. coli* LplA to detect whether the purified PdhD could be lipoylated *in vitro*. As shown in Figure 3B, PdhD was successfully modified by *E. coli* LplA *in vitro* in the presence of lipoic acid. These results suggest that the failure of Mhp-Lpl to modify PdhD was not because of structure collapse but because Mhp-Lpl cannot recognize this substrate, and a new enzyme must exist in *M. hyopneumoniae* to complete the lipoylation of PdhD.

To determine the enzyme modifying PdhD, BLASTp searches against the *M. hyopneumoniae* strain 232 genome (accession number: NC_006360) were carried out with the amino acid sequences of *E. coli* LplA (UniProt code: P32099) and *B. subtilis* LplJ (UniProt code: O07608), which are required for lipoic acid salvage. Apart from MHP_RS01680, previously reported as Mhp-Lpl by us (Zhu et al., 2020), we found that MHP_RS00640 (renamed it as Mhp-LplJ) shared 33.9% identity with *E. coli* LplA and 39% identity with *B. subtilis* LplJ. Mhp-LplJ may be a new lipoate-protein ligase in *M. hyopneumoniae.* Lipoate-protein ligases mostly consist of a large N-terminal domain and a small C-terminal domain. We first model the structure of the Mhp-LplJ protein using the Swiss-Model server with LplA of *Streptococcus pneumoniae* (*S. pneumoniae*) (PDBid: 1vqz) as the modeling template, which shows the highest reliability with a global model quality estimation (GMQE) value of 0.72. As expected, the Mhp-LplJ prediction model has a typical structure of lipoate-protein ligase, consisting of a large N-terminal domain (residues 1–245) and a small C-terminal domain (residues 252–336), and the two domains are linked by a short polypeptide chain (residues 246–252) (Figure 3C). The similarity between Mhp-LplJ and other lipoate-protein ligases, Mhp-Lpl, *B. subtilis* LplJ and *E. coli* LplA protein sequences, were further analyzed. The multiple sequence alignments showed that Mhp-LplJ contained the same conserved sequence motifs as *E. coli* LplA and *B. subtilis* LplJ (Supplementary Figure S1).

### Mhp-LplJ Can Catalyze the Lipoylation of GcvH and PdhD *in vitro*

**FIGURE 4:**
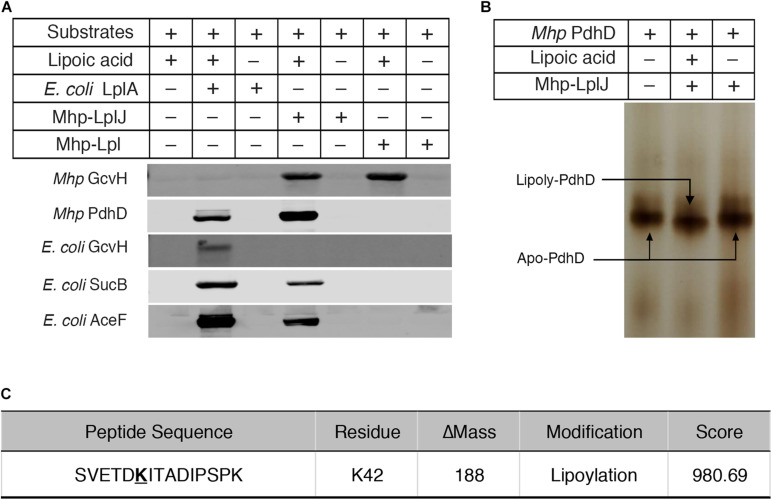
Functional characterization of Mhp-LplJ *in vitro*. **(A)** The lipoate ligase activities of Mhp-LplJ and Mhp-Lpl were identified using lipoate ligase assays *in vitro*, with *E. coli* LplA as the positive control. Purified *Mhp* GcvH, *Mhp* PdhD, GcvH of *E. coli* (*E. coli* GcvH), SucB of *E. coli* (*E. coli* SucB), and AceF of *E. coli* (*E. coli* AceF) were used as substrates. Lipoate modification of the substrate proteins was detected by western blot assays using Anti-LA. **(B)** Lipoate-modified PdhD catalyzed by Mhp-LplJ was analyzed using the gel shift assay. Loss of the positive charge at the lysine-binding site during lipoate modification resulted in faster migration on a native gel. **(C)** The lipoylation of PdhD was analyzed after chymotrypsin and trypsin digestion using LC-MS/MS assay. The lipoylated lysine residue of the peptide is in bold and underlined.

To characterize the enzymatic properties and compare the lipoate ligase activities of Mhp-LplJ with Mhp-Lpl *in vitro*, *M. hyopneumoniae* Mhp-LplJ, Mhp-Lpl and *E. coli* LplA proteins were expressed in the *E. coli* BL21 (DE3) strain and purified (Supplementary Figure S2A), apo lipoate-dependent proteins of *M. hyopneumoniae* (GcvH and PdhD) and *E. coli* (GcvH, dihydrolipoyllysine-residue succinyltransferase component of OGDH SucB encoded by *sucB*, and AceF encoded by *aceF*) were expressed in strain DE0626 in which the *lplA* and *lipB* genes were deleted to shut down lipoic acid metabolism and purified (Supplementary Figure S2B). We tested the lipoate ligase activities of Mhp-LplJ and Mhp-Lpl *in vitro* with *E. coli* LplA as a positive control. Mhp-Lpl lipoylates *M. hyopneumoniae* GcvH as previously reported; however, it does not lipoylate PdhD and three substrates of *E. coli* under the same conditions (Figure 4A). By contrast, Mhp-LplJ can lipoylate *M. hyopneumoniae* GcvH, PdhD, *E. coli* SucB and AceF, but cannot lipoylate *E. coli* GcvH. To further verify the lipoylation of PdhD catalyzed by Mhp-LplJ *in vitro*, a gel shift assay and liquid chromatography tandem mass spectrometry (LC-MS/MS) were carried out. Loss of the positive lysine charge upon lipoylation resulted in the more rapid migration of PdhD on native polyacrylamide gel electrophoresis (Figure 4B). The LC-MS/MS results further confirmed that the K_42_ of the PdhD protein is modified by lipoate attachment (Figure 4C and Supplementary Figure S3). These results indicated that Mhp-LplJ is a new lipoate-protein ligase responsible for the lipoate modification of PdhD in *M. hyopneumoniae*.

### Functional Characterization of Mhp-LplJ *in vivo*

**FIGURE 5:**
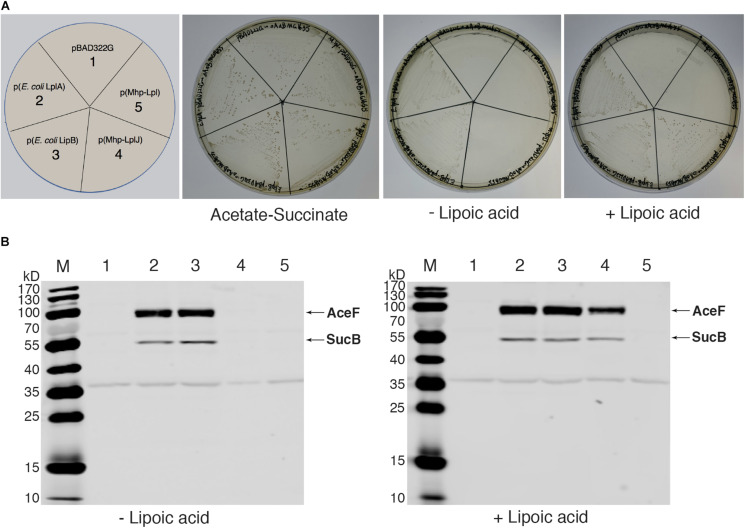
Complementation of an *E. coli* lipoyl-deficient strain with Mhp-LplJ and Mhp-Lpl. **(A)** Growth of strain DE0626 (MG1655 Δ*lplA* Δ*lipB*) transformed with pBAD322G-derived plasmids expressing the indicated genes. The complementation assays were performed on M9 minimal agar containing 0.2% L-arabinose. The control strains were the strain DE0626 transformed with the empty vector pBAD322G or derived plasmids expressing the indicated *E. coli* genes. The complemented strain DE0626 expressing *E. coli* LplA grew well on M9 minimal plate without lipoic acid because LplA could scavenge free octanoic acid from *E. coli* cytosol, and the octanoyl group was converted to lipoyl group under the catalysis of LipA. **(B)** Western blot analyses with Anti-LA to determine levels of lipoylation in complemented strains. The strains were grown for 24 h in Luria broth supplemented (+) or not (–) with lipoic acid. Lane M is the molecular weight ladder. The strains of lanes 1–5 corresponded to those in **(A)**. The protein of bands is indicated.

As there is no technique for knocking out genes in *M. hyopneumoniae* so far, complementation analysis could not be carried out in *M. hyopneumoniae.* To analyze the functions of Mhp-LplJ *in vivo*, we tested its ability to restore growth of the *E. coli* Δ*lplA* Δ*lipB* strain DE0626. In this strain, the lipoic acid salvage pathway and synthesis pathway were blocked, it was able to grow on M9 minimal agar plates only when the synthesis pathway is completed, or the salvage pathway is rebuilt with a plasmid encoding a lipoate-protein ligase at the presence of lipoic acid. As shown in Figure 5A, upon compensation with Mhp-LplJ, the model strain grew well in M9 minimal plate when lipoic acid was added. Mhp-Lpl, as a control group, could not restore the growth of strain DE0626 under the same conditions. These results indicated that Mhp-LplJ can catalyze the salvage pathway of lipoic acid and has a different function from Mhp-Lpl *in vivo*. Western blot assays further indicated that SucB and AceF of the strains that could grow on M9 plates are lipoylated (Figure 5B).

**FIGURE 6:**
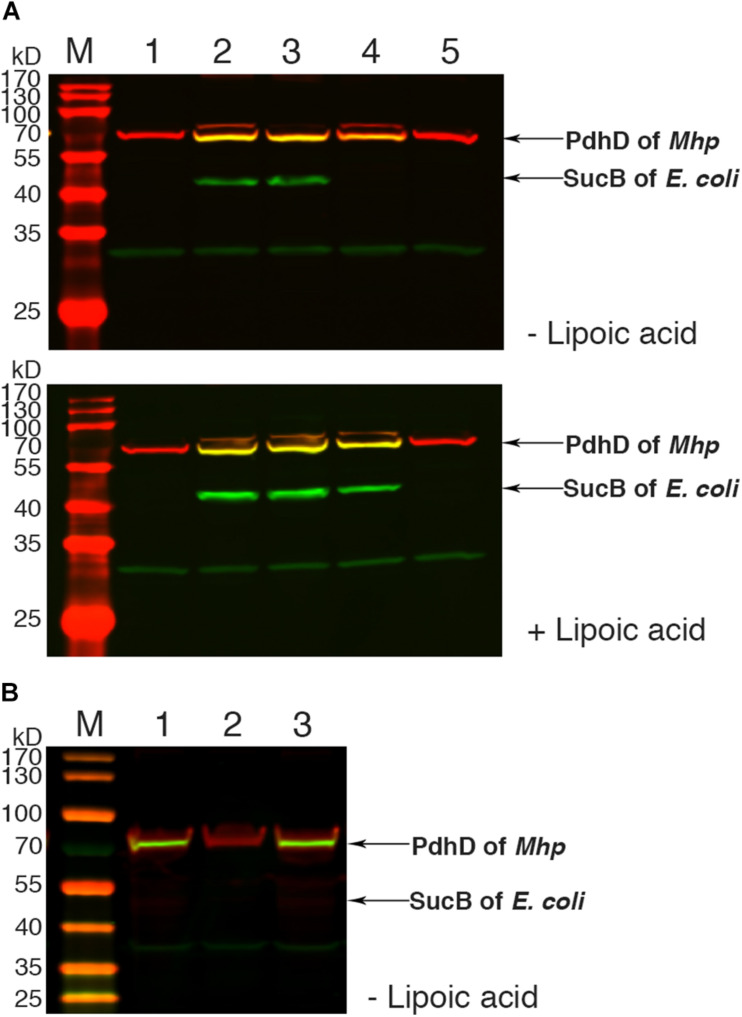
Functional analysis of Mhp-LplJ *in vivo*. **(A)** Western blot analyses of protein lipoylation in RE0403 (MG1655 Δ*aceF:pdhD* Δ*lplA* Δ*lipB*) complemented strains. Lanes 1–5, strain RE0403 expressing no enzyme, *E. coli* LplA, *E. coli* LipB, Mhp-LplJ, and Mhp-Lpl respectively. **(B)** Western blot analyses of protein lipoylation in RE0403-derived strains. Lane 1, RE0403 (MG1655 Δ*aceF:pdhD* Δ*lplA* Δ*lipB*) expressing Mhp-LplJ; lane 2, RE1011 (MG1655 Δ*aceF:pdhD* Δ*lplA* Δ*lipB* Δ*lipA*) expressing Mhp-LplJ; lane 3, RE1009 (MG1655 Δ*aceF:pdhD* Δ*lplA* Δ*lipB* Δ*gcvH*) expressing Mhp-LplJ. The total protein of bacteria was analyzed with western blot assays after culturing for 36 h in Luria broth supplemented (+) or not (–) with lipoic acid using both Anti-LA (green) and Anti-PdhD (red).

To further confirm the lipoate ligase activity of Mhp-LplJ on PdhD *in vivo*, the homologous *aceF* gene in the DE0626 genome was replaced with *pdhD* of *M. hyopneumoniae*. The strain was transformed with pBAD322G-derived plasmids, and the lipoylated proteins were analyzed using western blot assay. As shown in Figure 6A, Mhp-LplJ can catalyze the lipoylation of PdhD and SucB in the presence of lipoic acid. However, to our surprise, in the absence of lipoic acid, PdhD can also be lipoylated by Mhp-LplJ, whereas SucB cannot. To determine the proteins involved in the lipoylation of PdhD without free lipoic acid, we disrupted *lipA* and *gcvH*, which function in the lipoate synthesis pathway of other species (Miller et al., 2000; Christensen et al., 2011b), in the genome of RE0403 strain to generate strains RE1011 and RE1009, respectively. As shown in Figure 6B, *lipA* gene deletion prevented the lipoylation of PdhD in strain RE1011, while *gcvH* removal did not differ in strain RE1009. These results suggest that lipoyl synthase LipA cooperates with Mhp-LplJ to complete the lipoylation of PdhD in the absence of lipoic acid, while GcvH does not play any role in this pathway. Referring to the octanoate ligase activity of *E. coli* LplA (Hermes and Cronan, 2009), we hypothesized that Mhp-LplJ first scavenged free cytosolic octanoic acid from *E. coli*, and then LipA catalyzed the insertion of two sulfur atoms into the C-6 and C-8 positions of the octanoyl moiety bound to PdhD, converting the octanoyl moiety into lipoylated derivatives. We further verified the octanoate ligase activity of Mhp-LplJ by octanoate ligation assay *in vitro*. As shown in Supplementary Figure S4, attachment of octanoic acid to PdhD results in loss of a positive charge, which causes the modified PdhD to migrate more rapidly in gel shift assay. This result provided convincing evidence that Mhp-LplJ could able to catalyze the attachment of free octanoic acid to PdhD.

To identify the key residues of Mhp-LplJ, computational molecular docking was used to characterize the favored binding models of Mhp-LplJ with lipoic acid or lipoyl-AMP (Supplementary Figures S5A,B). The alignment of amino acid sequences showed that the residues of Mhp-LplJ forming the lipoic acid and lipoyl-AMP pockets are conserved with *E. coli* LplA and *B. subtilis* LplJ. We further identified the key residues of Mhp-LplJ using site-directed mutagenesis according to the favored binding models of Mhp-LplJ with lipoic acid or lipoyl-AMP, and the results implied that G^77^ and K^131^ are the key amino residues for Mhp-LplJ activity (Supplementary Figure S5C).

### Lipoic Acid Analogs Can Disrupt the Lipoate Scavenging Catalyzed by Mhp-LplJ and Arrest the Growth of *M. hyopneumoniae*

**FIGURE 7:**
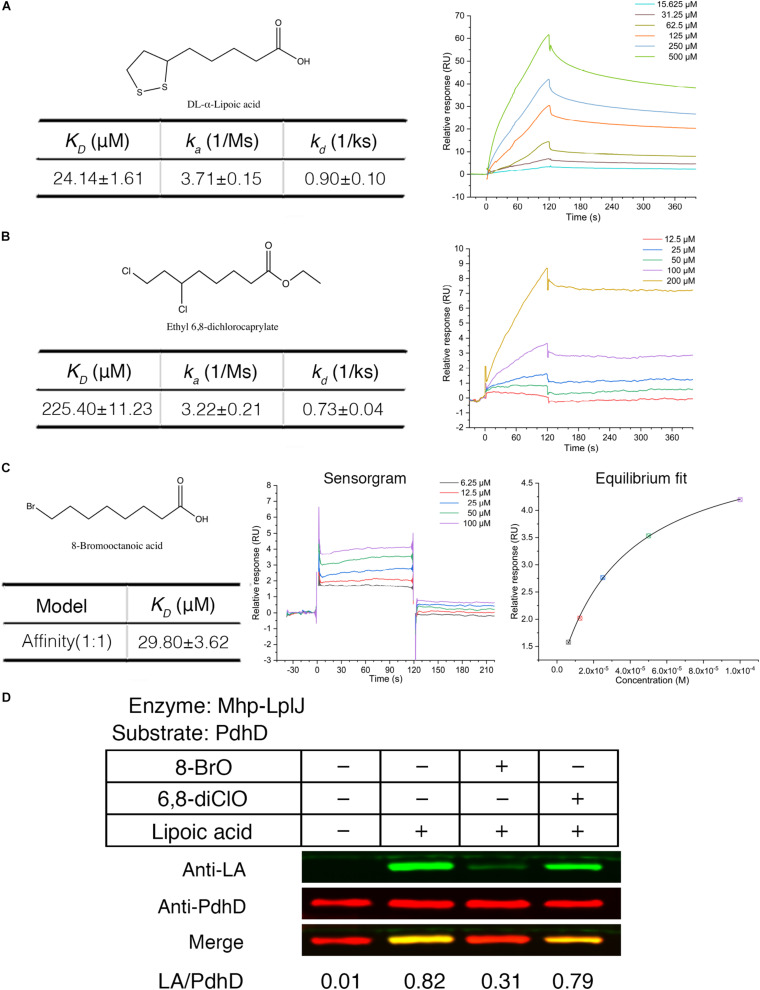
Lipoic acid analogs 8-BrO and 6,8-diClO can affect the lipoylation of PdhD catalyzed by Mhp-LplJ *in vitro*. Real-time interactions of lipoic acid and analogs binding to Mhp-LplJ were assessed by SPR. **(A)** Structure of lipoic acid and sensorgram for lipoic acid binding to Mhp-LplJ. **(B)** Structure of 6,8-diClO and sensorgram for 6,8-diClO binding to Mhp-LplJ. **(C)** Structure of 8-BrO, sensorgram and equilibrium fit for 8-BrO binding to Mhp-LplJ. **(D)** Lipoic acid analogs disturbed the lipoylation of PdhD catalyzed by Mhp-LplJ. In the lipoate ligation assay, lipoic acid and its analogs (1 mM) were added as indicated. The results were detected by western blot analysis using Anti-LA (green) and Anti-PdhD (red) after reaction.

Biotin (hexahydro-2-oxo-1H-thieno[3,4-d]imidazole-4-penta- noic acid, vitamin B_7_, vitamin H) and lipoic acid share many similarities in properties, metabolism and function. Biotin protein ligase (BPL) was proven to be a new drug target. Some biotin analogs have potential as antibacterial agents by inhibiting BPLs from *Staphylococcus aureus* (*S. aureus*), *E. coli*, *Mycobacterium tuberculosis* (*M. tuberculosis*), and *Homo sapiens*. To investigate whether lipoic acid analogs affect the function of Mhp-LplJ and the growth of *M. hyopneumoniae*, analogs 8-BrO and 6,8-diClO were selected for the tests. First, the binding characteristics of lipoic acid, 8-BrO and 6,8-diClO to Mhp-LplJ were analyzed by SPR. As shown in Figure 7, the sensorgrams of lipoic acid and 6,8-diClO are fitted to a single-site kinetics model, while the sensorgram of 8-BrO is suitable for the single-site affinity model. 8-BrO and lipoic acid exhibit similar equilibrium dissociation constants (*K*_*Ds*_) of 29.80 ± 3.62 μM and 24.14 ± 1.61 μM, respectively, whereas 8-BrO displays a quick association and disassociation pattern for Mhp-LplJ. 6,8-diClO shows a lower binding affinity to Mhp-LplJ, and the *K*_*D*_ value is one order of magnitude higher than that of lipoic acid (Figure 7B). Then, we determined the effects of these two lipoic acid analogs on Mhp-LplJ activity *in vitro*. Consistent with the measured binding affinity, 8-BrO with higher affinity has a more obvious interference effect on the lipoate ligase activity of Mhp-LplJ than 6,8-diClO (Figure 7D).

**FIGURE 8:**
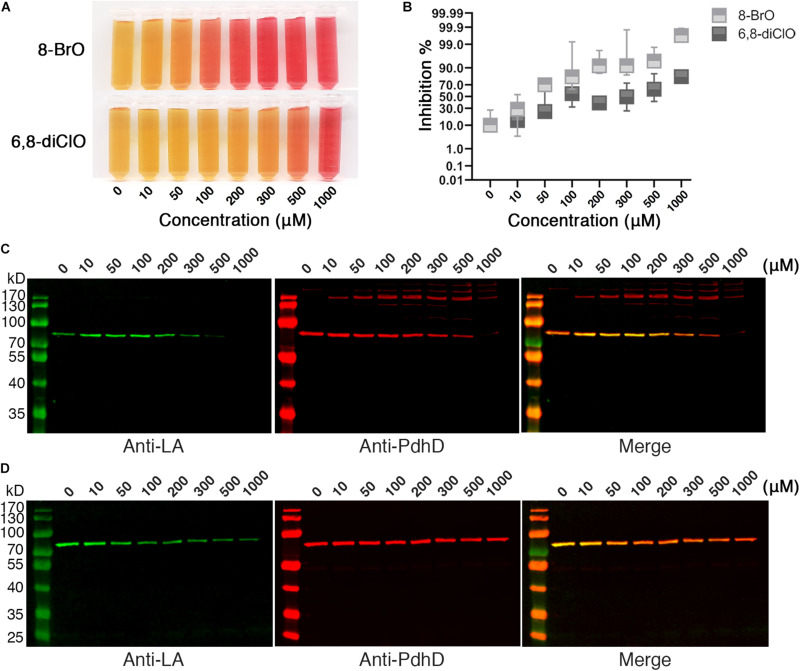
Effects of lipoic acid analogs on *M. hyopneumoniae* cultures. **(A)** Effects of lipoic acid analogs on *M. hyopneumoniae in vitro* growth. *M. hyopneumoniae* were cultured for 10 days in the presence of indicated concentrations of 8-BrO or 6,8-diClO. The color change of the medium (containing phenol red indicator) roughly reflects the bacterial concentration. **(B)** The inhibition rate of 8-BrO and 6,8-diClO at different concentrations. The number of viable *M. hyopneumoniae* was estimated by CCU assay. **(C)** Effect of 8-BrO at different concentrations on PdhD lipoylation. To collect enough cells for western blot assays, the samples with different concentrations of 8-BrO were cultured for another 10 days. Not enough *M. hyopneumoniae* were collected under a concentration of 1000 μM. **(D)** Effect of 6,8-diClO at different concentrations on PdhD lipoylation.

The attachment of lipoic acid is necessary for activation of PDH, and the activity of PDH affects the energy metabolism of microorganisms. We further investigated whether lipoic acid analogs that could disrupt lipoate ligase activity of Mhp-LplJ affect the growth of *M. hyopneumoniae*. *M. hyopneumoniae* was grown in mycoplasma medium supplemented with analogs at different concentrations. After culturing for an indicated time, the color of cultures presented a gradient change along the analog concentration (Figure 8A). The numbers of viable *M. hyopneumoniae* showed a negative correlation with the concentrations of lipoate analogs when determined with the CCU assay. As shown in Figure 8B, the lipoate analogs inhibit *M. hyopneumoniae* growth in a dose-dependent manner. Compared with 6,8-diClO, 8-BrO has a more obvious inhibitory effect. When the concentration of 8-BrO increased from 10 to 50 μM, the number of viable bacteria decreased sharply, and the inhibition rate increased from 28.57 to 70%. To verify that the growth inhibition of *M. hyopneumoniae* by the two analogs is correlated with the function disruption of Mhp-LplJ *in vivo*, we tested the lipoylation level of PdhD in these cultures. As shown in Figures 8C,D, the degree of lipoylation of PdhD was decreased with increasing analog concentration, but what puzzled us was that a certain number of different size bands appeared when 8-BrO was added. We also performed an inhibitory analysis of the two lipoate analogs in the *E. coli* strain RE1011. Similarly to the results found in *M. hyopneumoniae*, both analogs disrupted the enzymatic activity of Mhp-LplJ. However, multiple bands of PdhD also appeared when 8-BrO was added to the strain RE1011 (Supplementary Figure S6). To explain the multiple bands of PdhD, we further explored it through an *in vitro* lipoate ligation assay. As shown in Supplementary Figure S7, the PdhD bands with high-molecular weight cannot be probed when 8-BrO or Mhp-LplJ exists alone, but can only be detected when they are added to the reaction at the same time. Based on the above results, we speculate that the PdhD bands with high-molecular weight are due to the nucleophilic reactions of bromoalkanes, which leads to crosslinking of two proteins in the process of 8-BrO interfering with PdhD lipoylation catalyzed by Mhp-LplJ. The PdhD bands with high-molecular weight were analyzed by LC-MS and the results showed that both PdhD and Mhp-LplJ were present in these bands (data not shown).

The above results strongly suggest that the activity of Mhp-LplJ could be disrupted by lipoic acid analogs, and disruption of Mhp-LplJ function can inhibit the *in vitro* growth of *M. hyopneumoniae*.

## Discussion

Lipoic acid is a sulfur-containing cofactor that is essential for the function of several key enzymes involved in oxidative metabolism, including PDH, OGDH, BCOADH, and the glycine cleavage system, in most prokaryotic and eukaryotic organisms (Reed and Hackert, 1990; Cronan, 2016). Lipoate-protein ligases are responsible for the lipoate modification of these enzymes (Reed et al., 1958). We previously reported that Mhp-Lpl of *M. hyopneumoniae* is a member of the lipoate-protein ligase family and can catalyze the lipoylation of *M. hyopneumoniae* GcvH *in vitro* (Zhu et al., 2020). However, unexpectedly, Mhp-Lpl did not lipoylate PdhD, a lipoate-dependent subunit of *M. hyopneumoniae* (Figure 3A). According to this phenomenon, we found a novel lipoate-protein ligase, Mhp-LplJ, with a more complex function in *M. hyopneumoniae*. Mhp-LplJ can modify both GcvH and PdhD proteins of *M. hyopneumoniae.* Furthermore, although the gene *gcvH* does exist in *M. hyopneumoniae*, we did not find the genes encoding the other subunits of the Gcs. Strangely, the Anti-LA did not detect any the GcvH band in western blot analysis of *M. hyopneumoniae* total proteins (Figure 2). In some bacteria, the abundant expression of GcvH needs to be induced by glycine (Ramaswamy and Anthony, 2010). Whether glycine induction is required for expression of GcvH in *M. hyopneumoniae* needs further verification in the future. In Figure 3B, a 50 kD-sized band appeared under the PdhD protein when probing with Anti-LA. We are not sure what the band is. But we speculate that it may be related to *E. coli* LplA, because it also appeared in *E. coli* LplA-added group when the *E. coli* AceF as substrate in the original figure of Figure 4A (data not shown).

Like other mycoplasmas, *M. hyopneumoniae* has some adenosine triphosphate (ATP) protection mechanisms, including pyruvate roundhouse, which cope with the lack of a tricarboxylic acid cycle (TCA cycle). The pyruvate roundhouse pathway is closely correlated with the metabolism of mycoplasmas and may also play a role in the degradation of glucogenic and ketogenic amino acids (Razin et al., 1998). PDH catalyzes the first step of the pyruvate roundhouse, so PDH activity is essential for steady-state growth and metabolism of *M. hyopneumoniae*. In most prokaryotic species, the LDs of PDH are always located in the E2 subunit dihydrolipoamide acetyltransferase (PdhC) (Perham, 2000). There are a few exceptions, such as the cases in *Alcaligenes eutrophus* (Hein and Steinbüchel, 1994) and *Neisseria meningitidis* (Bringas and Fernandez, 1995), where LDs were found in E3 subunit PdhD, while in *Zymomonas mobilis*, LDs were detected in pyruvate dehydrogenase E1 component subunit alpha (PdhA) (Ute et al., 1999). Our results indicated that the LD of *M. hyopneumoniae* PDH is in the PdhD subunit (Figures 2, 4). This result is consistent with the prediction by Matic et al. (2003) that was made through sequence analysis of the *pdhCD* operon.

To date, two lipoate-protein ligases, Mhp-Lpl (Zhu et al., 2020) and Mhp-LplJ, have been identified in *M. hyopneumoniae.* It is uncommon for bacteria to have two lipoate-protein ligases responsible for the salvage pathway of lipoic acid metabolism. However, *Listeria monocytogenes* (*L. monocytogenes*) and *S. aureus* are the special cases (Keeney et al., 2007; Zorzoli et al., 2016). *L. monocytogenes* is a lipoate auxotroph bacterium and must rely on exogenous lipoic acid for growth (O’Riordan et al., 2003). There are two genes encoding lipoate-protein ligases in the *L. monocytogenes* genome, *lplA1* and *lplA2* (Keeney et al., 2007). Both LplA1 and LplA2 contribute to PDH lipoylation during extracellular growth, but only LplA1 is essential for intracellular growth and virulence. And the lipoyl ligation is specific for GcvH in *L. monocytogenes* (Christensen et al., 2011a). In *S. aureus*, there are not only two lipoate protein ligases LplA1 and LplA2, but also two H protein homologous to GcvH and GcvH-L. In the salvage pathway of lipoic acid, free lipoic acid is first attached to GcvH and GcvH-L under the catalysis of *S. aureus* LplA1, then transferred to E2 subunits by LipL, while *S. aureus* LplA2 could transfer the cofactor to GcvH-L as well as the E2 subunits of other lipoate-requiring enzymes directly (Laczkovich et al., 2018). Of the two lipoate-protein ligases of *M. hyopneumoniae*, Mhp-LplJ shows lipoate ligase activity on both GcvH and PdhD, while Mhp-Lpl specifically catalyzes free lipoic acid attachment to GcvH (Figure 4A). How the two ligases work together to support the growth of *M. hyopneumoniae* is unclear, and it is interesting to further explore the functional correlation between Mhp-LplJ and Mhp-Lpl in *M. hyopneumoniae*.

Given the inability to edit the genome precisely in *M. hyopneumoniae*, the function of Mhp-LplJ could not be analyzed in *M. hyopneumoniae*. Here, we used an *E. coli* system to analyze the *in vivo* function of Mhp-LplJ as done for the *Streptomyces coelicolor* lipoate-protein ligase (Cao and Cronan, 2015) and the human octanoyl transferase LipT2 (Cao et al., 2018c). This system provides an ideal environment for the functional analysis of heterologous lipoate-protein ligases *in vivo* if these ligases cannot be analyzed in their native organisms. Some researchers also used a lipoylation-deficient *E. coli* strain transformed with a plasmid expressing a candidate lipoate-protein ligase and an expression plasmid holding a lipoylation substrate to perform the cell-based lipoylation assay (Afanador et al., 2014, 2017). In this study, we replaced the *aceF* gene of the *E. coli* lipoylation-deficient strain with *M. hyopneumoniae pdhD* and obtained the derived strain RE0403. Compared to the expression vector, the expression level of the target gene integrated into the genome was closer to the physiological state. In addition, we studied the *in vivo* modification of PdhD by Mhp-LplJ based on strain RE0403. Our results showed that in the absence of lipoic acid, Mhp-LplJ could complete the lipoylation of PdhD with cooperation of LipA (Figure 6B). We further demonstrated that Mhp-LplJ also has octanoate ligase activity. In the lipoic acid synthesis pathway of *E. coli*, LplA can scavenge free octanoic acid to unlipoylated apo proteins, and then LipA performs sulfur insertion to form a lipoyl group (Miller et al., 2000; Hermes and Cronan, 2009). However, we failed to find the genes encoding enzymes participating in the lipoic acid synthesis pathway using a BLAST search with the *E. coli* and *B. subtilis* homologs against the *M. hyopneumoniae* genomes. Even though it was found here that Mhp-LplJ possesses octanoate ligase activity, we do not know whether or how Mhp-LplJ performs this function in *M. hyopneumoniae.* More research needs to be done to answer these questions in the future.

Previous reports indicated that suppressors of essential metabolic enzymes could be developed as new classes of antibiotics. For example, BPLs have been studied for a few years as drug targets for new antibiotics (Costa et al., 2012; Feng et al., 2016a, b). Studies have shown that biotin analogs, as inhibitors of BPL, have significant antibacterial activity against *M. tuberculosis* (Duckworth et al., 2011; Bockman et al., 2015), *E. coli* (Xu and Beckett, 1994; Brown and Beckett, 2005), *S. aureus* (Costa et al., 2012; Pendini et al., 2013; Paparella et al., 2014), and so on. Lipoic acid and biotin share many similarities in function and metabolic pathways (Cronan, 2014). However, lipoate-protein ligase has rarely been reported as an antibacterial target of prokaryotic microorganisms. Here, for the first time, we found that lipoic acid analogs 8-BrO and 6,8-diClO can disturb the lipoylation of PdhD catalyzed by Mhp-LplJ to different degrees *in vitro* (Figure 7D) and *in vivo* (Figures 8C,D). In contrast to 6,8-diClO, in the inhibition analysis with lipoic acid analogs 8-BrO, many bands appeared when probed with Anti-PdhD (Figure 8C). Growth inhibition assays show that both 8-BrO and 6,8-diClO can obviously inhibit the cell growth of *M. hyopneumoniae in vitro* (Figures 8A,B). Although we cannot deduce that the inhibitory effect of 8-BrO and 6,8-diClO on the *M. hyopneumoniae* growth is simply caused by the functional disruption of Mhp-LplJ, it is confirmed that functional disruption of Mhp-LplJ is partially responsible for the growth inhibition because the lipoylation of PdhD is decreased in the inhibited *M. hyopneumoniae.* In addition, we speculate that the interference effect of analogs on Mhp-LplJ activity had two possible ways: one is that the lipoic acid analogs occupied the lipoic acid binding pocket of Mhp-LplJ competitively; the other way was that Mhp-LplJ could transfer the analogs instead of lipoic acid to the lipoic acid binding site of lipoate-dependent proteins. However, regardless of the mechanism, our current experimental results indicate that Mhp-LplJ has potential to be explored as a novel drug target of *M. hyopneumoniae*. We could further design and develop lipoic acid analogs based on structural and biochemical studies on Mhp-LplJ for curing *M. hyopneumoniae* infection.

Together, the above results indicate that Mhp-LplJ is a novel lipoate-protein ligase of *M. hyopneumoniae* that possesses a different biological function from Mhp-Lpl, whose function had been previously identified by our laboratory (Zhu et al., 2020). Mhp-LplJ plays a vital role in lipoic acid metabolism of *M. hyopneumoniae*, and disruption of Mhp-LplJ function is fatal to *M. hyopneumoniae.*

## Data Availability Statement

The datasets presented in this study can be found in online repositories. The names of the repository/repositories and accession number(s) can be found in the article Supplementary Material.

## Ethics Statement

The animal study was reviewed and approved by Institutional Animal Care and Use Committee of Harbin Veterinary Research Institute.

## Author Contributions

JJ performed the experiment and wrote the manuscript. HC completed the structural modeling and molecular docking. NW, KZ, and HL helped construct the plasmids and produce the monoclonal antibodies. DS and JX helped perform the analysis with constructive discussions. HL designed the experiments, wrote and revised the manuscript. All the authors contributed to the article and approved the submitted version.

## Conflict of Interest

The authors declare that the research was conducted in the absence of any commercial or financial relationships that could be construed as a potential conflict of interest.
