# Supplementary material for: A Novel Lipoate-Protein Ligase, Mhp-LplJ, Is Required for Lipoic Acid Metabolism in Mycoplasma hyopneumoniae
Source: Front Microbiol. 2021 Jan 18;11:631433. doi: 10.3389/fmicb.2020.631433 (PMC7873978; doi:10.3389/fmicb.2020.631433)
Supplement: Supplementary file 1 [file Data_Sheet_1.PDF]

Supplementary Material

Supplementary Figures

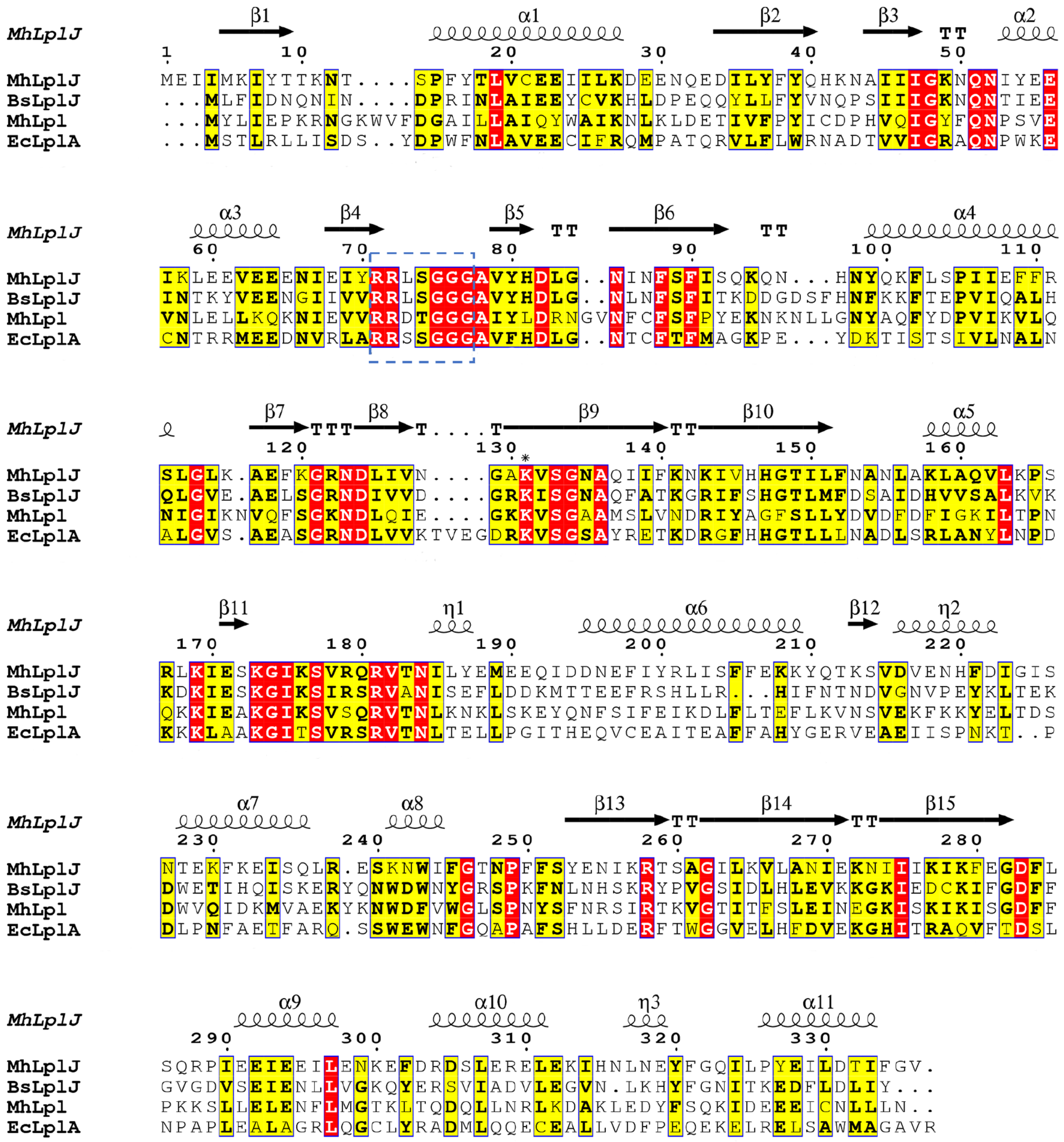

Figure S1. Amino acid sequence alignments of Mhp-LplJ (MhLplJ), *B. subtilis* LplJ (BsLplJ), Mhp-Lpl (MhLpl) and *E. coli* LplA (EcLplA). Conserved residues are marked with a red background, and similar residues are marked with yellow. The Mhp-LplJ secondary structure is shown above the aligned sequences. The key catalytic site, lysine residue, is marked with an asterisk. And lipoate binding motif "RRXXGGG" is circled in a blue dotted frame.

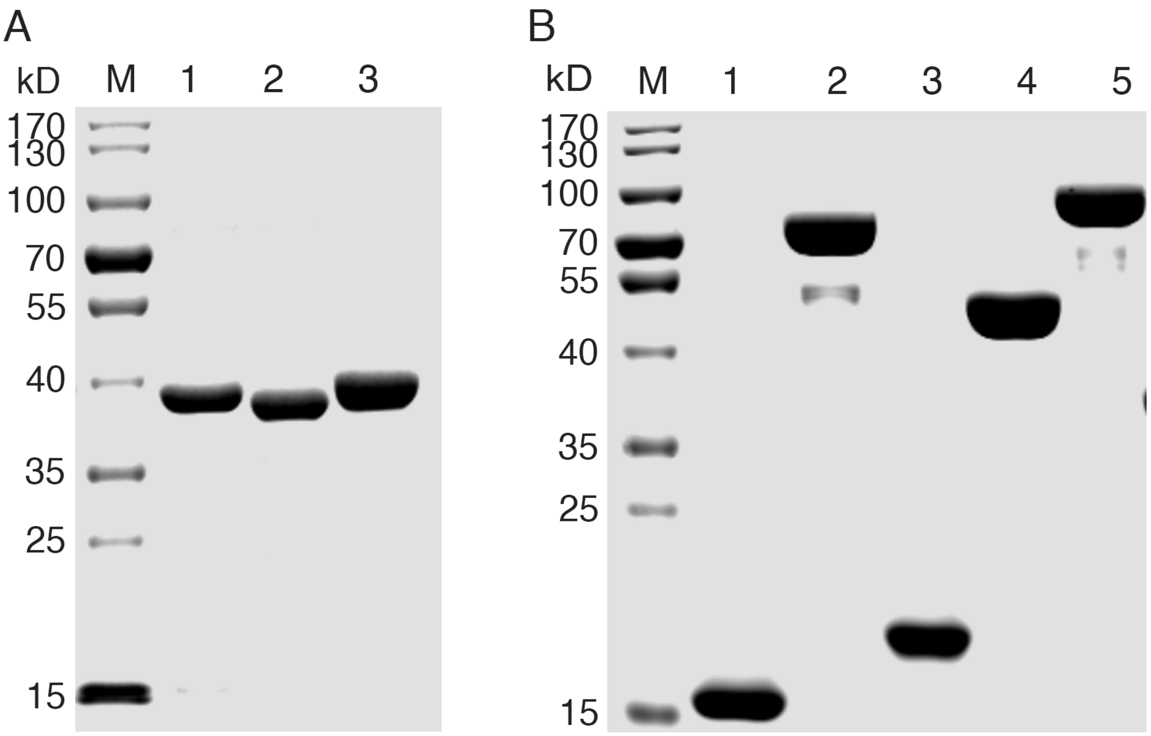

Figure S2. SDS-PAGE analysis of purified proteins. A) Purification of lipoate-protein ligases expressed in the *E. coli* BL21 (DE3) strain as described in the *Experimental Procedures*. Lane M is the prestained molecular weight ladder; lane 1, purified Mhp-LplJ; lane 2, purified Mhp-Lpl; lane 3, purified LplA of *E. coli*. B) Purification of apo lipoate-requiring substrates expressed in *E. coli* lipoic acid auxotroph strain DE0626. Lane M is the prestained molecular weight ladder, and lanes 1-5 are purified apo-GcvH of *M. hyopneumoniae*, apo-PdhD of *M. hyopneumoniae*, apo-GcvH of *E. coli*, apo-SucB of *E. coli* and apo-AceF of *E. coli*, respectively.

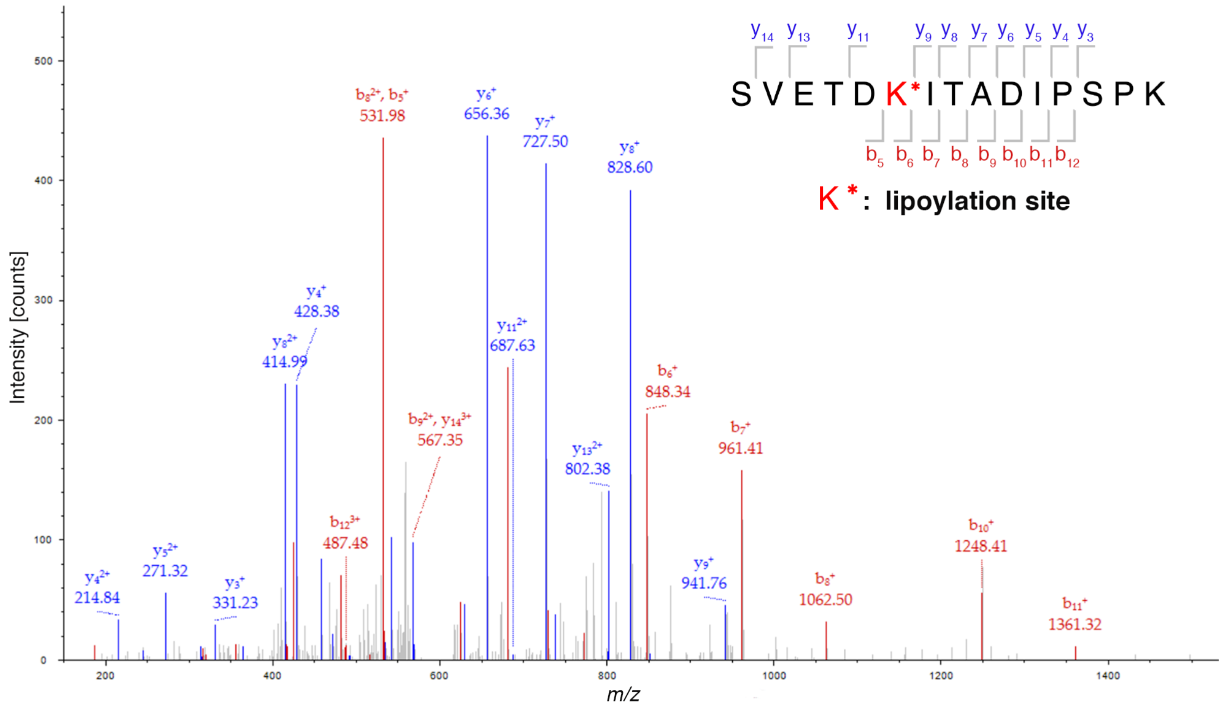

Figure S3. Analysis of lipoyl-PdhD using mass spectrometry. Nano-LC-LTQ-Orbitrap XL MS/MS analysis of lipoyl-PdhD after trypsin digestion. The detected peaks (main panel) correspond to the predicted peptides (inset), where red corresponds to observed N-terminal peptide fragments and blue corresponds to observed C-terminal peptide fragments. K* indicates Lys_{42}, which is the lipoate attached site.

Mhp PdhD	+	+	+
Mhp-LplJ	–	+	+
Octanoic acid	+	–	+

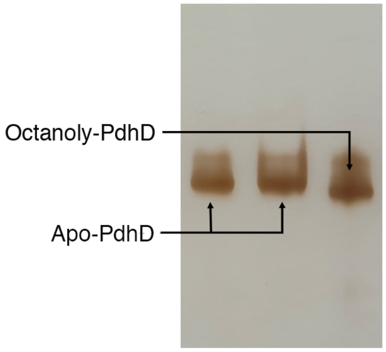

Figure S4. Octanoate ligase activity analysis of Mhp-LplJ using gel shift assay. The reaction was performed as the lipoate ligation assay in vitro. Octanoic acid was added to reactions instead of lipoic acid. Attachment of octanoic acid to PdhD results in loss of a positive charge, which causes the modified PdhD to migrate more rapidly in native gel electrophoresis.

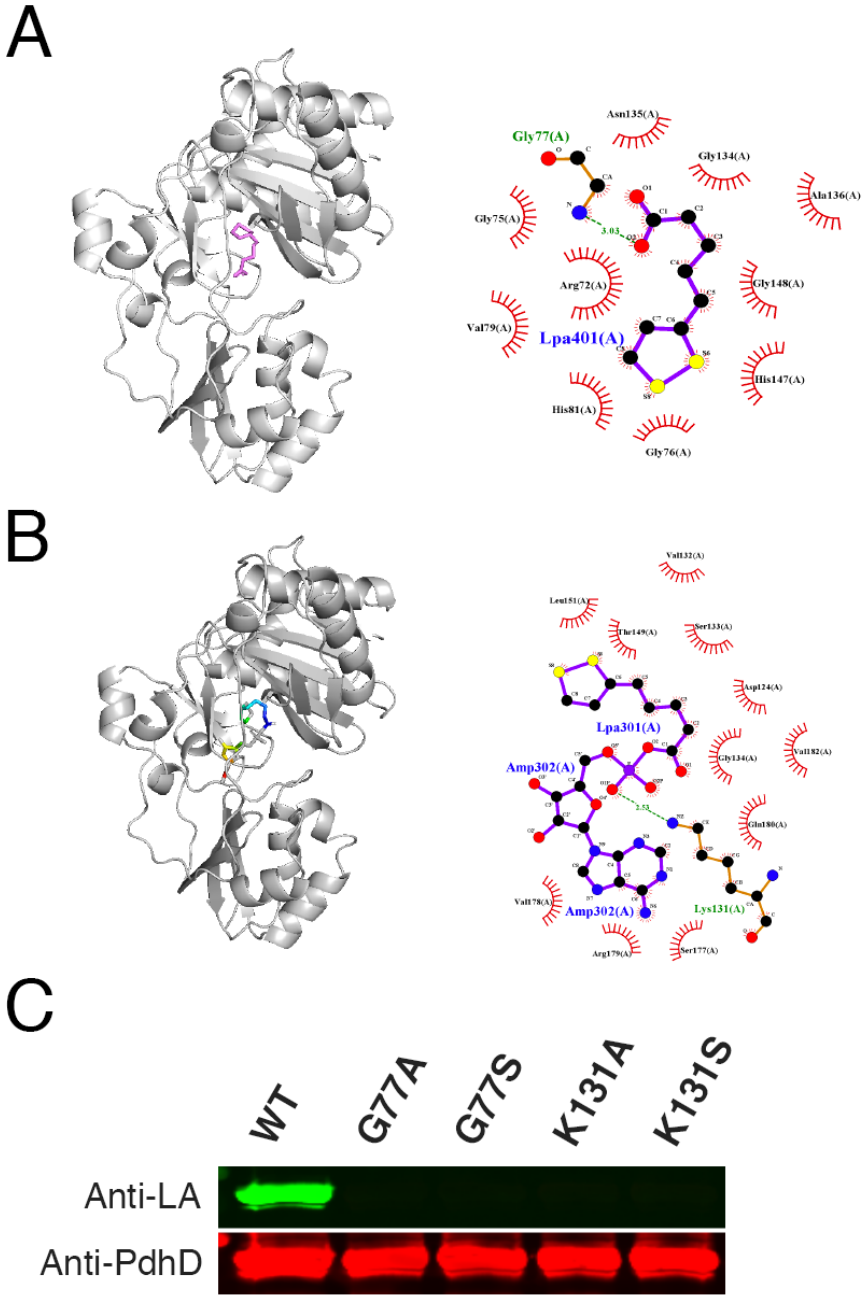

Figure S5. Analysis of the active sites of Mhp-LplJ. A) Modeling and pattern of the Mhp-LplJ bond with lipoic acid (LA). B) Modeling and pattern of the Mhp-LplJ bond with lipoyl-AMP. C) Gly^{77} and Lys^{131} at the active site of Mhp-LplJ were mutated into both Ala and Ser. The lipoate ligase activity of the mutated proteins was analyzed by lipoate ligation assays with PdhD as the substrate, and western blot assays were used to detect the lipoate modification of PdhD with Anti-LA (green) and Anti-PdhD (red).

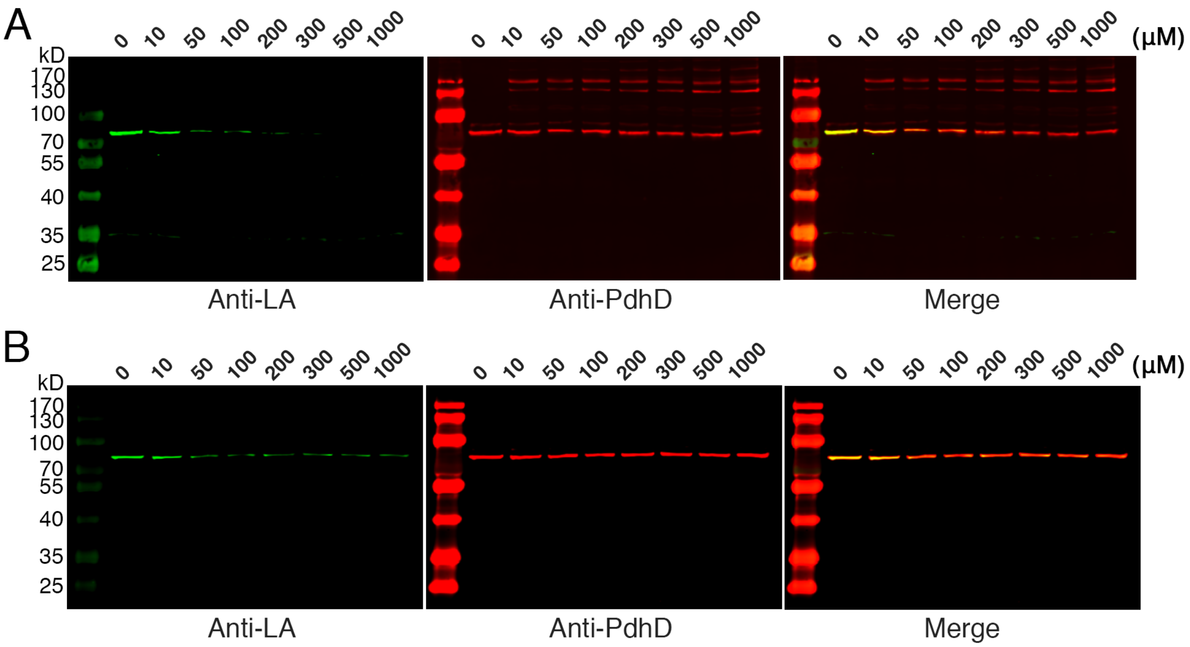

Figure S6. Inhibition of Mhp-LplJ lipoylation activity by lipoic acid analogs based on model organism strain RE1011 (MG1655 Δ*aceF::pdhD* Δ*lplA* Δ*lipB* Δ*lipA*). Strain RE1011 transformed with plasmid pBG003 encoding Mhp-LplJ was incubated at 37°C in LB medium containing 10 ng/mL lipoic acid, 0.2% (v/v) L-arabinose, and different concentrations of lipoic acid analogs. Isovolumetric DMSO was added as a negative control. The optical density (OD_{600}) of all samples was measured after 24 h of culture. All samples collected the same amounts of viable bacteria, and the lipoylation levels of PdhD were analyzed by western blot assays with Anti-LA (green) and Anti-PdhD (red). A) Effect of 8-BrO on PdhD lipoylation catalyzed by Mhp-LplJ in model organisms. B) Effect of 6,8-diClO on PdhD lipoylation catalyzed by Mhp-LplJ in model organisms.

Lanes	1	2	3	1	2	3	1	2	3
8-BrO	−	+	+	−	+	+	−	+	+
Mhp-LplJ	+	−	+	+	−	+	+	−	+
Lipoic acid	+	+	+	+	+	+	+	+	+
PdhD	+	+	+	+	+	+	+	+	+

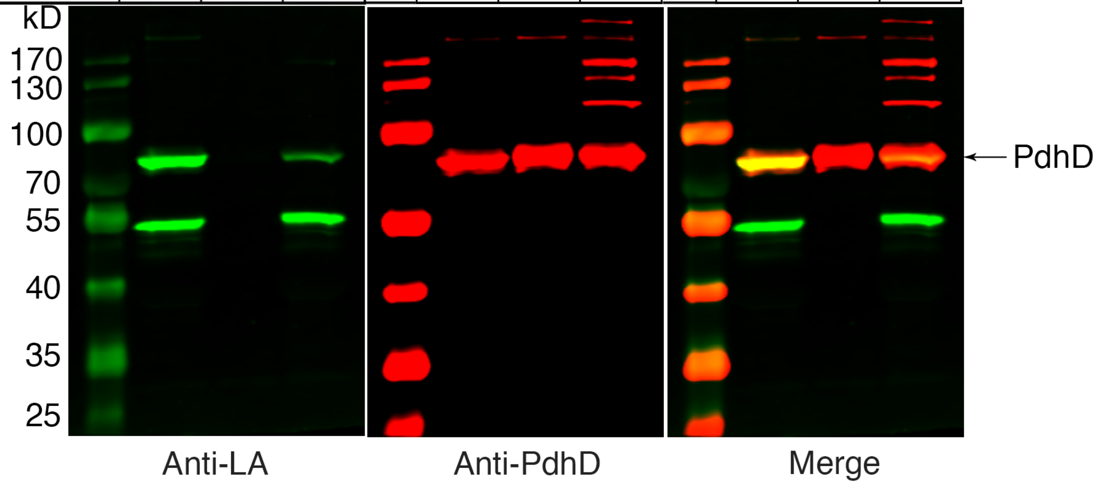

Figure S7. The effect of 8-BrO on PdhD was analyzed by lipoate ligation assay in vitro. The components added to the lipoate ligation reaction are shown in the table. The final concentrations of 8-BrO and lipoic acid are 1mM. After incubation at 37°C for 3 h, the reactions were analyzed by SDS-PAGE followed by western blot using Anti-LA (green) and Anti-PdhD (red). These results show that 8-BrO alone will not affect PdhD (Lane 2), and the larger bands recognized by Anti-PdhD appeared only when Mhp-LplJ exists at the same time (Lane 3).
